# Determination of the full-genome sequence of hepatitis E virus (HEV) SAAS-FX17 and use as a reference to identify putative HEV genotype 4 virulence determinants

**DOI:** 10.1186/1743-422X-9-264

**Published:** 2012-11-08

**Authors:** Yumin Zhu, Xiaoming Yu, Fenfen Huang, Ruisong Yu, Shijuan Dong, Fusheng Si, Yuanshu Zhang, Zhen Li

**Affiliations:** 1 Institute of Animal Science and Veterinary Medicine, Shanghai Academy of Agricultural Sciences, Shanghai, 201106, China; 2 Shanghai Key Laboratory of Agricultural Genetics and Breeding, Shanghai, 201106, China; 3 Veterinary College, Nanjing Agricultural University, Nanjing, Jiangsu, 210095, China; 4 College of Animal Science and Technology, Jiangxi Agricultural University, Nanchang, Jiangxi, 330000, China

**Keywords:** Hepatitis E virus, Disease severity determinant, Subtype, Genotype, Zoonosis

## Abstract

**Background:**

Four major genotypes of hepatitis E virus (HEV), the causative agent of hepatitis E, have so far been recognized. While genotypes 3 and 4 are both zoonotic, the disease symptoms caused by the latter tend to be more severe. To examine if specific nucleotide/amino acid variations between genotypes 3 and 4 play a role in determining the severity of hepatitis E disease, the complete genome of one swine HEV genotype 4 isolate, SAAS-FX17, was determined and compared with other genotype 4 and genotype 3 genomes to identify putative HEV genotype 4 virulence determinants.

**Results:**

A total of 42 conformable nt/aa variations between genotype 3 and 4 HEVs were detected, of which 19 were proposed to be potential disease severity determinants for genotype 4 strains.

**Conclusions:**

One potential determinant was located in each of the 5'-UTR and 3'-UTR, 3 and 12 within ORF1 and ORF2 respectively, and 2 in the junction region.

## Background

Hepatitis E virus (HEV) is an important human pathogen in many regions of the world, and is the causative agent of acute hepatitis, a disease spread mainly through fecal contamination of water supplies or food [1]. HEV is the sole member of the genus *Hepevirus* of the family Hepeviridae [2]. The virus is a single-stranded positive-sense RNA virus containing a short 5' untranslated region (UTR), three open reading frames (ORF1-3), and a 3' UTR [3]. ORF1 encodes non-structural proteins involved in viral replication, ORF2 encodes a structural protein comprising the virion capsid, and ORF3, which overlaps ORF2, is required for viral egress from infected cells [4].

Four major HEV genotypes and several subtypes within each genotype have been identified in mammalian species [5]. Genotypes 1 and 2 have been isolated from humans only and genotypes 3 and 4 are zoonotic [6]. Evidence is accumulating to indicate that different HEV genotypes are associated with disease symptoms of differing severity. For example, genotypes 3 and 4 appear to be less virulent for humans compared with genotypes 1 and 2 [7]. Furthermore, disease symptoms caused by genotype 4 are reported to be more severe compared with genotype 3 infections [8-10], and genotype 4 viral loads recorded in a co-infected patient were also higher [11].

In order to identify specific nucleotides/amino acids influencing the severity of HEV infections, we have now determined the full genome sequence of swine HEV strain, SAAS-FX17, which was previously classified as a genotype 4, sub-type (i) virus [12]. Potential determinants responsible for the observed variations in the severity of disease symptoms caused by genotype 3 and 4 strains were then identified by comparison with the corresponding sequences of 56 genotype 4 and 56 genotype 3 HEV’s retrieved from GenBank.

## Materials and methods

### Fecal specimens and extraction of RNA

Swine HEV, strain SAAS-FX17, was isolated from a fecal specimen collected in 2008 from a pig farm located in a Shanghai suburb. Sample treatment and total RNA extraction procedures were described previously [13].

### Reverse transcription and PCR

All the non-terminal reverse transcripts (RTs) were synthesized with the SuperScript™ III First-strand Synthesis System (Invitrogen, USA) using an external antisense primer, and the first-strand cDNA was used immediately for PCR.

Ten sets of specific external and internal primer pairs (see Additional file 1: Table S1) were used to amplify the entire viral genome. Nucleotide (nt) sequences at the 5' and 3' ends were determined with the SMART^TM^ Rapid Amplification of cDNA Ends (RACE) cDNA Amplification Kit (Clontech Laboratories, Japan) according to the manufacturer’s instructions.

### Sequence analysis

PCR products were purified and ligated into a pJET 1.2/blunt cloning vector (Fermentas). At least three positive clones were selected at random and sequenced (Sangon Biotech Shanghai Co., Ltd) in both directions using an ABI model 3730 automatic DNA sequencer (ABI, CA, USA).

Sequence assembly was accomplished and percent identity values were calculated using Lasergene (version 7.10; DNAstar). Sequence alignments were generated by Clustal-W (version 1.8). Phylogenetic trees were constructed by the neighbor-joining method with the aid of the MEGA 4.0 software package. Genetic distances were calculated by using the Kimura two-parameter method. One thousand re-samplings of the data were used to calculate branch percentage values. Secondary structures in the 5' UTR were predicted with the mfold program [14].

### Nucleotide sequence accession number

**Figure 1:**
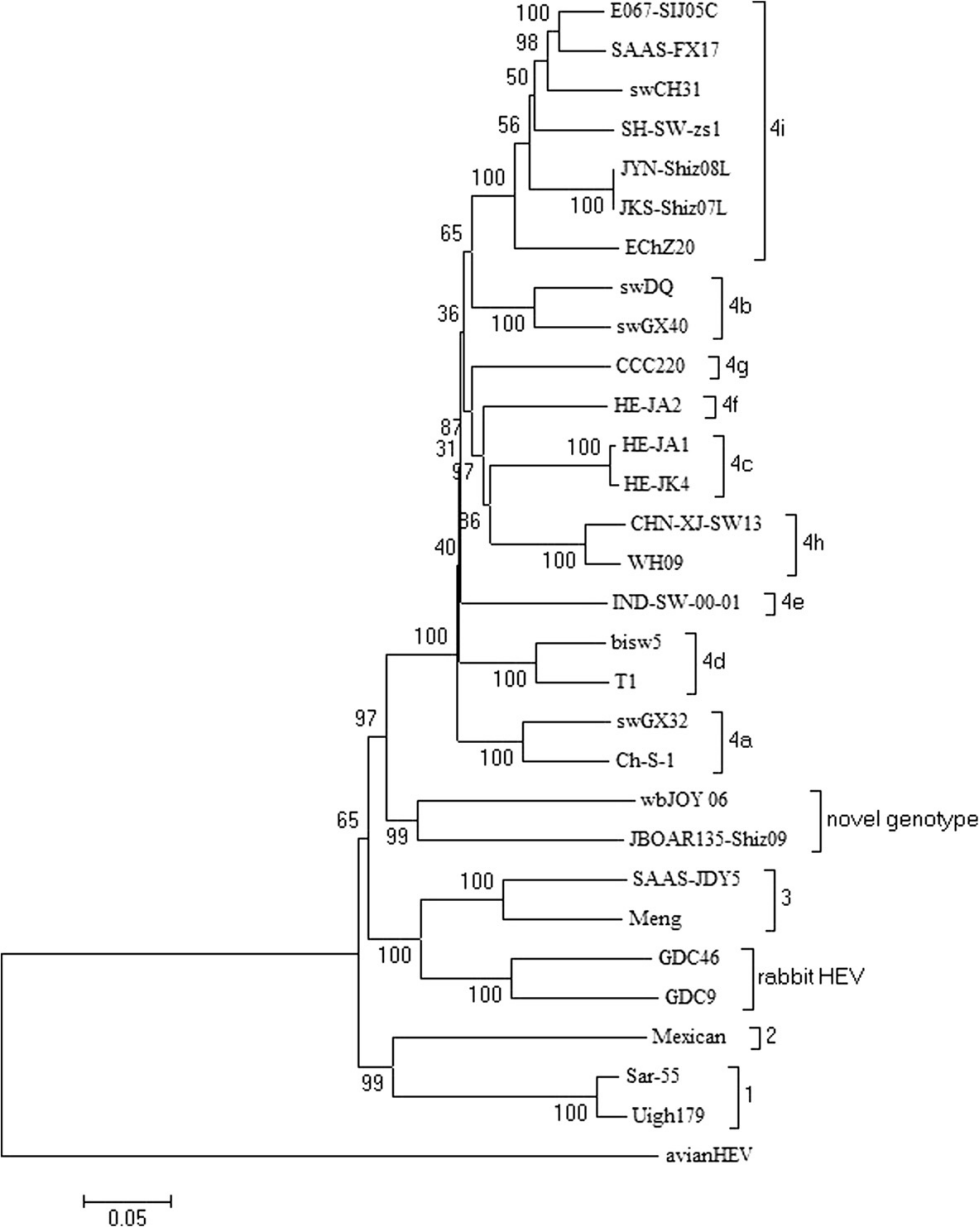
**Phylogenetic tree depicting the genotypic/subgenotypic status of 30 HEV isolates based on the full length genomic sequence.** Internal node numbers indicate the bootstrap values as a percentage of trees obtained from 1000 replicates.

The full genomic nucleotide sequence of strain SAAS-FX17 has been deposited in the GenBank database under accession number JF915746. Accession numbers and classifications of the 120 HEV reference strains are shown in Additional file 2: Table S2. Of these, 30 complete HEV genome sequences were selected at random in order to construct the genotype/subtype phylogenetic tree (Figure 1). All nt and amino acid (aa) comparisons are made with reference to strain SAAS-FX17.

## Results

### Genome organization of swine HEV strain SAAS-FX17

The full-length genome of strain SAAS-FX17 consisted of 7262 nt, excluding the poly (A) tail at the 3' terminus, and contained three ORFs. The genome was organized into a 5' UTR extending from nt 1–26, ORF1 from nt 27–5135 (5109 nt), ORF2 from 5174–7156 (1983 nt), and a smaller ORF3 (which overlapped ORF2) from 5166–5504 (339 nt). The 77 nt 3' UTR extended from 7157 to 7233 and was followed by a poly (A) tail of 29 A residues.

### Phylogenetic analysis

The nt sequence of SAAS-FX17 was 74.5-75.7% similar to all recorded HEV genotypes 1–3 and was 83.7-94.8% identical with the genome sequences of all reported genotype 4 HEVs. Consistent with an earlier classification [12], SAAS-FX17 was confirmed as belonging to subtype 4i within genotype 4 (Figure 1). Sequence similarity with other viruses within this subgroup was 90.6-94.8%, and 84.0-85.6% with members of the other genotype 4 sub-groupings (4a-4h).

The SAAS-FX17 genome is most closely related to that of a human HEV strain, E067-SIJ05C, derived from an acute hepatitis E patient in Japan, who had traveled to Shanghai prior to the onset of disease symptoms. These two isolates shared nt similarities of 94.8%, 94.6%, 95.1% and 97.6% in terms of the full-length genome, ORF1, ORF2 and ORF3, respectively.

### 5' UTR and 3' UTR analyses

**Figure 2:**
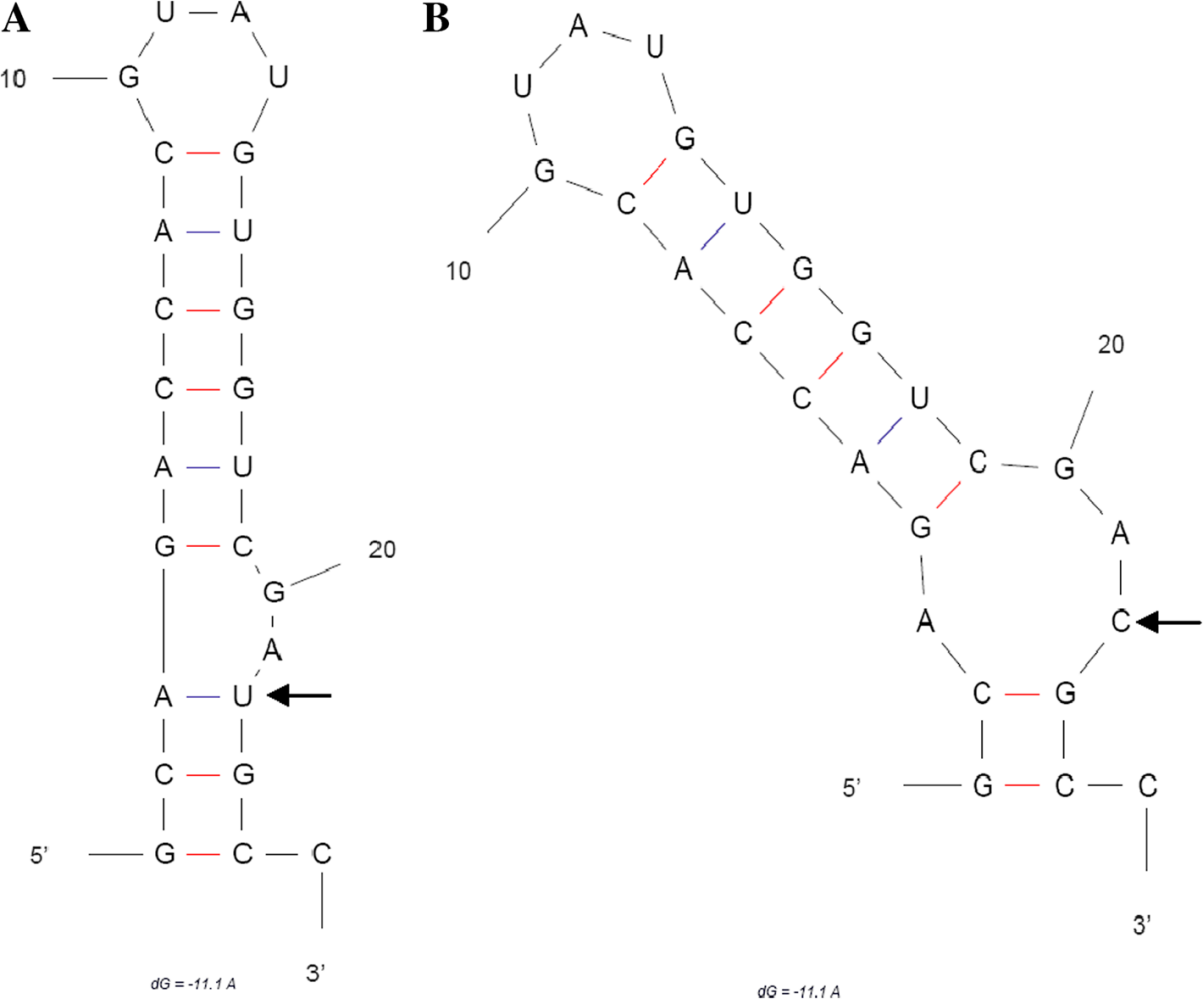
Secondary structures of the 5'-UTR region of HEV genotypes 3 (A) and genotypes 4 (B), as predicted by the mfold program. Arrows indicates the nucleotide variation between the different genotypes.

The 5' UTR of SAAS-FX17 consisted of 26 nt and contained one additional G at the extreme 5' terminus compared with the majority of genotype 4 strains. One unique nt substitution (C23T) was identified in the second loop structure of the 5' UTR of genotype 4 HEV strains which, as seen in Figure 2, contained eight nts compared with six for genotype 3 strains.

SAAS-FX17 contained a 3' UTR consisting of 77 nt excluding the poly (A) tail but including five additional nts (ACTGGT) at the terminus. 3' UTRs of all genotype 4 strains contained an initial TTTATT sequence as opposed to TTAATT in the 3' UTRs of genotype 3 strains.

### Analysis of ORFs

#### ORF1

The ORF1 of SAAS-FX17 consisted of 5109 (27–5135) nts capable of encoding a protein of 1702 aa. The nucleotide and amino acid sequences were 82.5-94.6% and 93.1-98.2% identical, respectively to the corresponding sequences of other genotype 4 isolates.

Excluding the hypervariable region (HVR), 12 genotype 4 specific aa substitutions were identified in the ORF1 (Table 1). Two aa variations were evident in the HVR, the initial conserved sequence of which was VSGFSSCFSP in genotype 4 strains compared with TSGFSSDFSP in genotype 3 strains (Table 1).

**Table 1:** Specific amino acid substitutions/insertions/deletions in the ORF1-3 regions of HEV genotype 4 strains

**Region**	**Amino acid variation and site**
ORF1	Substitutions: Q486E, R491L, E494D, A501E, F502P/L/E, E503A/V, S505L/V/I/F, D508S, P509G, A510T/S, T/A524H/Y/T, D1574N
HVR	Substitutions: T707V, D713C
ORF2	Substitutions: I66V, P/S67V/F/L, T/A68S/P, A/T69Q, T/A70S/P, P/S97A/T/V, A114S, T/A146S, I147V, T149S/A, S161N, S324T, S/G326T, K330R, G509A, C580A, N587S, S590A
ORF3	2 aa insertions (5P, 84Q) , 1 aa substitution (A35V) and 1 aa deletion (aa residue 68)

#### ORF2

The ORF2 of SAAS-FX17 consisted of 1983 nts capable of encoding a protein of 660 aa. The nucleotide and amino acid sequences were 86.7-95.1% and 93.9-99.1% identical, respectively to the corresponding sequences of other genotype 4 isolates. Comparison of the ORF2 proteins in the two HEV genotypes revealed a total of 18 aa substitutions (Table 1).

#### ORF3

The ORF3 of SAAS-FX17 consisted of 345 nts, encoding 114 aa. The nucleotide and amino acid sequences were 91.3-98.8% and 90.3-97.7% identical, respectively to the corresponding sequences of other genotype 4 isolates. The ORF3-encoded protein of genotype 4 strains contained two aa insertions (5P, 84Q) and one aa deletion (aa residue 68), an additional aa overall compared with genotype 3 viruses. Furthermore, there was an a substitution (A35V) when compared with genotype 3 (Table 1).

#### Junction region (JR)

**Figure 3:**
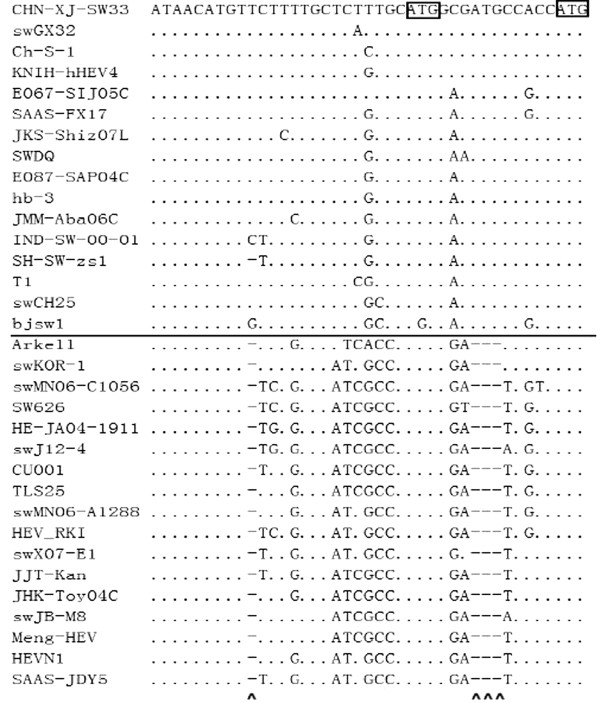
**Alignment of nucleotide sequences comprising the junction region (JR) of genotype 3 strains (below the line) and genotype 4 strains (above the line).** The first and second boxed ATG triplets constitute the ORF2 and ORF3 start codons, respectively. Deletions are indicated by dashes, identical nucleotides by dots, and insertion sites by ^. The strains were selected to represent all the genomic variations within the JR region of genotypes 3 and 4 HEV.

The JR[15] of SAAS-FX17 consisted of 38 bp, which is identical in length to most other genotype 4 strains but 4 bp longer than the corresponding region of genotype 3 strains. These additional 4 bp were due to 4 nt insertions at sites 10, 30, 31 and 32 of the JR. The space length (distance between the ORF2 and ORF3 initiation sites) was 11bp and 8bp for genotypes 4 and 3, respectively (Figure 3).

## Discussion

Since 2000, genotype 4 HEV has replaced genotype 1 as the dominant cause of hepatitis E in China [16-18]. Recently, Zhu *et al*. [12] identified a novel virus belonging to subtype 4i, the same subtype to which SAAS-FX17 has been assigned. In view of the close relationship between SAAS-FX17 and strain E067-SIJ05C, derived from an acute hepatitis E patient in Japan who had traveled to Shanghai before the onset of disease symptoms, it appears HEV strains belonging to this subtype may undergo zoonotic transmission.

Zhang *et al*. [19] previously suggested that the 5' UTR of the viral genome may play an important role in replication and/or translation. Other researchers reported that the 5' UTR and a conserved 58 nt region within ORF1 were likely to fold into conserved stem-loop and hairpin structures, which were postulated to be important for HEV RNA replication [20]. In the present study, a unique nt substitution, C23T, was identified within the putative stem-loop structure of the 5' UTR, which resulted in a change in the size of the second loop. Previous studies have shown that nt variations in the central portion of the 5' UTR may influence the severity of type A hepatitis [21]. However, although a potential virulence determinant for genotype 4 HEV, the impact of C23T on HEV disease symptoms remains to be established.

The 3' UTR and an adjacent region of the HEV genome form a putative stem-loop structure that affects the binding of recombinant viral RNA dependent RNA polymerase (RdRp) and initiation of RNA synthesis [22]. Graff *et al*. [23] showed that a seemingly minor change caused by a mutation at nt 7106, which eliminated one putative base pair within the stem-loop, significantly inhibited RNA replication, and the magnitude of virus replication could be the reason for the severity difference of HEV. Therefore, based on these previous research findings, a single nt difference (T3A) identified between the 3' UTR sequences of HEV genotypes 3 and 4 also represents a putative virulence determinant.

HEV ORF1 encodes a large nonstructural protein with several putative functional motifs [24]. Our data derived from sequence comparisons of this region in HEV genotypes 3 and 4 revealed 12 specific aa substitutions, 11 of which occurred in the protease motif. However, since no functional activity relating to disease severity has so far been attributed to this enzyme, these substitutions are unlikely to have a role as virulence determinants. The remaining substitution was located in the RdRp motif, which is essential for genomic RNA replication. In addition, two aa variations were recorded in the conserved initiation motifs of the HVRs of genotype 3 and 4 strains. Virus attenuation that accompanied complete deletion of this region of ORF1 led to the suggestion that the HVR played a biological role in HEV pathogenesis [25]. Therefore, we propose that these two variations together with the substitution in the RdRp motif are potential candidates for virulence variation in type 4 HEV.

ORF2 encodes the viral capsid protein, including a signal peptide (aa 1–22) involved in the translocation of the protein from the endoplasmic reticulum [26], and an arginine-rich domain (aa 23–111) that may be involved in RNA encapsidation [27]. Córdoba *et al*. [28] recently verified that mutations within the latter domain contributed to virus attenuation. Our data comparing HEV genotypes 3 and 4 identified six specific aa substitutions within this region, five of which (aa residues 66–70) were continuous. This entire sequence motif may represent a single putative virulence determinant [16], implying the possible existence of two such determinants in the entire arginine-rich region. Three structural domains have been defined within the C-terminus of the HEV capsid: S (residues 118–313), P1 (residues 314–453) and P2 (residues 454–606), which function in forming the capsid shell, binding of the virus to host cell receptors, and antigenicity, respectively [29,30]. Single or multiple variations in the aa sequences of the capsid or envelope proteins resulted in attenuated viral phenotypes [31,32] and, more recently, Córdoba *et al*. [28] verified that HEV attenuation was also linked to mutations in the P1 domain. Our study revealed four, three and four specific substitutions in the S, P1 and P2 domains, respectively of genotype 4 HEV. Each might constitute individual putative virulence determinants although the proximate substitutions at positions 146–147 could represent a single influencing factor.

ORF3 protein is essential for virion release from HEV infected cells [33]. However, it remains unclear if differences in the length of the ORF3 regions of HEV genotypes 3 and 4, or specific aa variations in the encoded proteins, influence the severity of the respective clinical symptoms.

The Junction Region (JR) denotes the genome segment between the stop codon of ORF1 and the putative initiation codon of ORF2, in which a bicistronic subgenomic mRNA encodes both ORF2 and ORF3 proteins of HEV [15]. Cao *et al*. [34] demonstrated that nt mutations or a mutation in the stem-loop structure formed within the JR significantly inhibited HEV replication. Furthermore, Shukla *et al*. [35] reported that the distance between the initiation codons of ORF2 and ORF3 affected initiation preferences. Therefore, nt mutations and distance variation between the ORF2 and ORF3 initiation codons within the JR of genotypes 3 and 4 may constitute strong candidates for determinants of disease severity. Although four nt insertions at sites 10, 30, 31 and 32 of the JR were identified in this study, the contiguous insertions at positions 30–32 may represent a single putative virulence determinant.

## Conclusions

A total of 42 nt/aa variations between HEV genotypes 3 and 4 were identified. Not all may constitute potential virulence determinants since several observed contiguous nt/aa variations, which are often responsible for a single mutation incident [16,36], may each constitute a single determinant. Based on this conjecture, the 42 nt/aa variations represented 19 potential determinants. However, the possibility of additional putative determinants existing among the 42 variations cannot be excluded.

## Competing interests

The authors declare that there are no competing interests.

## Authors’ contributions

YZ carried out the full-genome sequence determination. YZ and ZL participated in the sequence alignment and drafted the manuscript. XY, FH, RY, SD, FS and YZ participated in parts of the study. All authors read and approved the final manuscript.

## Supplementary Material

Additional file 1 **Table S1.** Genomic position and nucleotide sequence of oligonucleotide primers for PCR. The genomic position refers to strain SAAS-FX17 (JF915746) adopted in this study. ES, EA, IS and IA denote ‘external sense’, ‘external antisense’, ‘internal sense’ and ‘internal antisense’, respectively. Click here for file

Additional file 2 **Table S2.** Genotype, strain designation and GenBank accession numbers of 120 HEV reference strains employed in this study. Click here for file
