# Supplementary material for: Determination of the full-genome sequence of hepatitis E virus (HEV) SAAS-FX17 and use as a reference to identify putative HEV genotype 4 virulence determinants
Source: Virol J. 2012 Nov 8;9:264. doi: 10.1186/1743-422X-9-264 (PMC3546022; doi:10.1186/1743-422X-9-264)
Supplement: Additional file 1 — Table S1. Genomic position and nucleotide sequence of oligonucleotide primers for PCR. The genomic position refers to strain SAAS-FX17 (JF915746) adopted in this study. ES, EA, IS and IA denote ‘external sense’, ‘external antisense’, ‘internal sense’ and ‘internal antisense’, respectively. [file 1743-422X-9-264-S1.doc]

Supplementary Table 1: Genomic position and nucleotide sequence of oligonucleotide primers for PCR. The genomic position refers to strain SAAS-FX17 (JF915746) adopted in this study. ES, EA, IS and IA denote ‘external sense’, ‘external antisense’, ‘internal sense’ and ‘internal antisense’, respectively.

| Primer | Genomic position | Primer sequence (5'-3') | Product size (bp) |
| --- | --- | --- | --- |
| 5'EA | 1016-1040 | CGAGCAGCAAAAAGCCTGGTCATCA | 747 |
| 5'IA | 723-747 | GGATCCAGGCGCGCAGAATAGACAC |
| ES1 | 617-634 | ACTTCCTCCTGGCACCTA | 552 |
| EA1 | 1201-1219 | AACCTCTTCATTCCTTTCG |
| IS1 | 628-646 | GCACCTATCATACCACATC |
| IA1 | 1162-1179 | ACCTCTGATGACAAATGG |
| ES2 | 1097-1114 | TGCTAACGAAGGCTGGAA | 1433 |
| EA2 | 2568-2585 | GGTGTAGGCTGCAAACCC |
| IS2 | 1118-1135 | CTCCGAGGATGCACTGAC |
| IA2 | 2533-2550 | CAGCGGGATCAAATGACT |
| ES3 | 2408-2425 | TGATGGCTCGAAGGTGTA | 701 |
| EA3 | 3814-3232 | GCAGGGATTTGATTAGGAT |
| IS3 | 2438-2457 | TTTTGAGTCCGACTGTACTT |
| IA3 | 3120-3138 | CAATAACAACCCTACGACC |
| ES4 | 2209-2227 | TTGAGACTGACACGCCTAC | 1108 |
| EA4 | 4301-4317 | CAATAGCACGGAACCAG |
| IS4 | 3122-3141 | TCGTAGGGTTGTTATTGATG |
| IA4 | 4210-4229 | TTCGCCAGTTGTAAACTTAT |
| ES5 | 3974-3991 | GACTCTTGTCGGTAGGTA | 828 |
| EA5 | 5074-5092 | GGTTTAACTGTTTCGGTAA |
| IS5 | 4047-4065 | AATCATTTTATCCCCGAGC |
| IA5 | 4856-4874 | CCCAGCAAACCTAACAACA |
| ES6 | 4634-4652 | TGTCTGGAATATGGCAGTT | 1147 |
| EA6 | 5848-5866 | ATCGGTAGAGGTGATGGAA |
| IS6 | 4657-4675 | CCCACTGTTATGAGTTCCG |
| IA6 | 5785-5803 | GGAGATGGATATGGCGTAC |
| ES7 | 5717-5736 | TATGCTCAGTATCGCGTTGT | 986 |
| EA7 | 6901-6920 | TGGAAATACAGACTCGGTGA |
| IS7 | 5733-5750 | TTGTCCGTGCCACTATTC |
| IA7 | 6701-6718 | GTCCAGGGAACGAGAAAC |
| 3'ES | 6698-6722 | GGGGTTTCTCGTTCCCTGGACTGGT | 221 |
| 3'IS | 7042-7066 | CGATGATTTCTGCCCTGAGTGCCGT |
