# Supplementary material for: Determination of the full-genome sequence of hepatitis E virus (HEV) SAAS-FX17 and use as a reference to identify putative HEV genotype 4 virulence determinants
Source: Virol J. 2012 Nov 8;9:264. doi: 10.1186/1743-422X-9-264 (PMC3546022; doi:10.1186/1743-422X-9-264)
Supplement: Additional file 2 — Table S2. Genotype, strain designation and GenBank accession numbers of 120 HEV reference strains employed in this study. [file 1743-422X-9-264-S2.doc]

Genotype, strain designation and GenBank accession numbers of 120 HEV reference strains employed in this study

| **Genotype** | **Strain designation** | **GenBank Accession No.** | **Genotype** | **Strain designation** | **GenBank Accession No.** |
| --- | --- | --- | --- | --- | --- |
| 1 | Uigh179 | D11093 | 4 | JAK-Sai | AB074915 |
|  | SAR-55 | M80581 |  | JKK-Sap | AB074917 |
| 2 | Mexican strain | M74506 |  | HE-JI4 | AB080575 |
| 3 | swJ570 | AB073912 |  | JSN-Sap-FH | AB091395 |
|  | JKN-Sap | AB074918 |  | swJ13-1 | AB097811 |
|  | JMY-Haw | AB074920 |  | HE-JA1 | AB097812 |
|  | HE-JA10 | AB089824 |  | HE-JK4 | AB099347 |
|  | JJT-Kan | AB091394 |  | CCC220 | AB108537 |
|  | JBOAR1-Hyo04 | AB189070 |  | JSF-Tot03C | AB193176 |
|  | JDEER-Hyo03L | AB189071 |  | JYN-Sap01C | AB193177 |
|  | JMO-Hyo03L | AB189072 |  | JYN-Nii02L | AB193178 |
|  | JSO-Hyo03L | AB189073 |  | JKO-ChiSai98C | AB197673 |
|  | JTH-Hyo03L | AB189074 |  | JYI-ChiSai01C | AB197674 |
|  | JYO-Hyo03L | AB189075 |  | JSN-Sap-FH02C | AB200239 |
|  | HEVN1 | AB246676 |  | HE-JF3 | AB220971 |
|  | HE-JA04-1911 | AB248520 |  | HE-JF4 | AB220972 |
|  | swJ8-5 | AB248521 |  | HE-JF5 | AB220973 |
|  | swJ12-4 | AB248522 |  | HE-JA2 | AB220974 |
|  | swMN06-A1288 | AB290312 |  | HE-JA19 | AB220975 |
|  | swMN06-C1056 | AB290313 |  | HE-JA28 | AB220976 |
|  | JIO-Sai97L | AB291951 |  | HE-JA36 | AB220977 |
|  | JIY-Tot05L | AB291952 |  | HE-JA37 | AB220978 |
|  | JSO-Oki05L | AB291953 |  | HE-JA41 | AB220979 |
|  | JSS-Oka04L | AB291954 |  | HEVN2 | AB253420 |
|  | JSW-Kyo-FH06L | AB291955 |  | JTC-Kit-FH04L | AB291959 |
|  | JYM-Tot04L | AB291956 |  | JYK-Tok03C | AB291964 |
|  | JYU-Oki04L | AB291957 |  | HRC-HE14C | AB291965 |
|  | JNH-Ehi04L | AB291958 |  | JST-KitAsa04C | AB291966 |
|  | JTK-Kag06C | AB291960 |  | JKO-Aba-FH06C | AB291967 |
|  | JMH-Osa04C | AB291961 |  | JMM-Aba06C | AB291968 |
|  | JHK-Toy04C | AB291962 |  | E087-SAP04C | AB369688 |
|  | JRM-Toy05C | AB291963 |  | E067-SIJ05C | AB369690 |
|  | JE03-1760F | AB301710 |  | JKS-Shiz07L | AB521805 |
|  | E116-YKH98C | AB369687 |  | JYN-Shiz08L | AB521806 |
|  | E097-OSA05C | AB369691 |  | HE-Aichi-C1 | AB602439 |
|  | JIO-swJ19-1 | AB443623 |  | WBjgf_08_1 | AB602440 |
|  | JIO-swJ19-2 | AB443624 |  | T1 | AJ272108 |
|  | JIO-swJ19-5 | AB443625 |  | swCH25 | AY594199 |
|  | JIO-swJ19-7 | AB443626 |  | IND-SW-00-01 | AY723745 |
|  | JIO-swJ19-8 | AB443627 |  | SWDQ | DQ279091 |
|  | swJB-E10 | AB481226 |  | SWCH31 | DQ450072 |
|  | swJB-M8 | AB481228 |  | Ch-S-1 | EF077630 |
|  | swJR-P5 | AB481229 |  | SH-SW-zs1 | EF570133 |
|  | HEV-US1 | AF060668 |  | SWGX32 | EU366959 |
|  | Meng-HEV | AF082843 |  | swGX40 | EU676172 |
|  | JRA1 | AP003430 |  | swCH189 | FJ610232 |
|  | Arkell | AY115488 |  | KNIH-hHEV4 | FJ763142 |
|  | swX07-E1 | EU360977 |  | CHN-XJ-SW33 | GU119960 |
|  | Thai-swHEV07 | EU375463 |  | CHN-XJ-SW13 | GU119961 |
|  | TLS25 | EU495148 |  | WH09 | GU188851 |
|  | SW626 | EU723512 |  | bjsw1 | GU206559 |
|  | SW627 | EU723513 |  | hb-3 | GU361892 |
|  | SWP6 | EU723514 |  | bisw5 | HM152568 |
|  | SWP7 | EU723515 |  | Echz20 | HM439284 |
|  | swKOR-1 | FJ426403 |  | TW6196E | HQ634346 |
|  | swKOR-2 | FJ426404 |  | SAAS-FX17 | JF915746 |
|  | SAAS-JDY5 | FJ527832 | Avian HEV | Avian HEV | AY535004 |
|  | CU001 | FJ653660 | Novel genotype | JBOAR135-Shiz09 | AB573435 |
|  | HEV_RKI | FJ956757 |  | WbJOY_06 | AB602441 |
|  |  | | Rabbit genotype | GDC9 | FJ906895 |
|  |  |  |  | GDC46 | FJ906896 |
